# Bereavement and type 1 diabetes in childhood: a register-based cohort study in Sweden

**DOI:** 10.1007/s00125-024-06340-z

**Published:** 2024-12-19

**Authors:** Mona-Lisa Wernroth, Beatrice Kennedy, Katja Fall, Diem Nguyen, Awad I. Smew, Per-Ola Carlsson, Bodil Svennblad, Catarina Almqvist, Tove Fall

**Affiliations:** 1 Molecular Epidemiology, Department of Medical Sciences, Uppsala University, Uppsala, Sweden; 2 Uppsala Clinical Research Center, Uppsala University, Uppsala, Sweden; 3 Clinical Epidemiology and Biostatistics, School of Medical Sciences, Örebro University, Örebro, Sweden; 4 Department of Medical Epidemiology and Biostatistics, Karolinska Institutet, Stockholm, Sweden; 5 Department of Perioperative Medicine and Intensive Care, Karolinska University Hospital, Stockholm, Sweden; 6 Department of Medical Cell Biology, Uppsala University, Uppsala, Sweden; 7 Transplantation and Regenerative Medicine, Department of Medical Sciences, Uppsala University, Uppsala, Sweden; 8 Medical Epidemiology, Department of Surgical Sciences, Uppsala University, Uppsala, Sweden; 9 Pediatric Allergy and Pulmonology Unit at Astrid Lindgren Children’s Hospital, Karolinska University Hospital, Stockholm, Sweden

**Keywords:** Bereavement, Cohort, Family caregiver, Psychological stress, Type 1 diabetes

## Abstract

**Aims/hypothesis:**

The potential impact of childhood bereavement—a severe psychological stressor—on childhood type 1 diabetes development remains unclear. Here, we aimed to bridge this knowledge gap and assess whether bereavement characteristics influenced any impact.

**Methods:**

We conducted a register-based cohort study encompassing 3,598,159 children born in Sweden between 1987 and 2020. Childhood bereavement was defined as the death of a biological mother, father or sibling. Diagnosis of type 1 diabetes in childhood (<18 years) was ascertained through the National Patient Register. We applied a Cox proportional hazards regression model to investigate the impact of childhood bereavement on type 1 diabetes, while adjusting for potential confounders (including parental type 1 diabetes status, country of birth and demographic characteristics).

**Results:**

During follow-up, 86,226 children (2.4%) lost a family member, and 18,817 children (0.52%) were diagnosed with type 1 diabetes (median age at onset 9.1 years). We did not detect any overall association between childhood bereavement and type 1 diabetes (adjusted HR 1.04; 95% CI 0.93, 1.17). We found no influence of age at loss, cause of death, familial relationship to the deceased, and time since loss.

**Conclusions/interpretation:**

In this large population-based Swedish study, we observed no evidence supporting a link between childhood bereavement and type 1 diabetes.

**Graphical Abstract:**

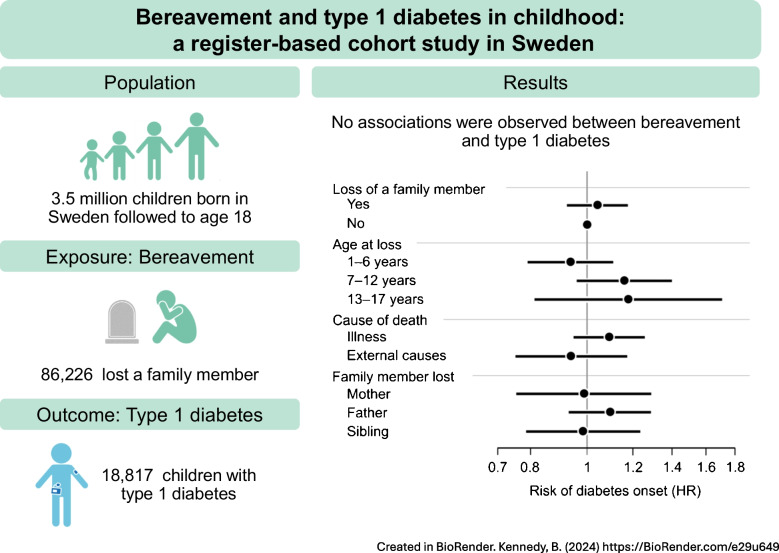

**Supplementary Information:**

The online version contains peer-reviewed but unedited supplementary material available at 10.1007/s00125-024-06340-z.


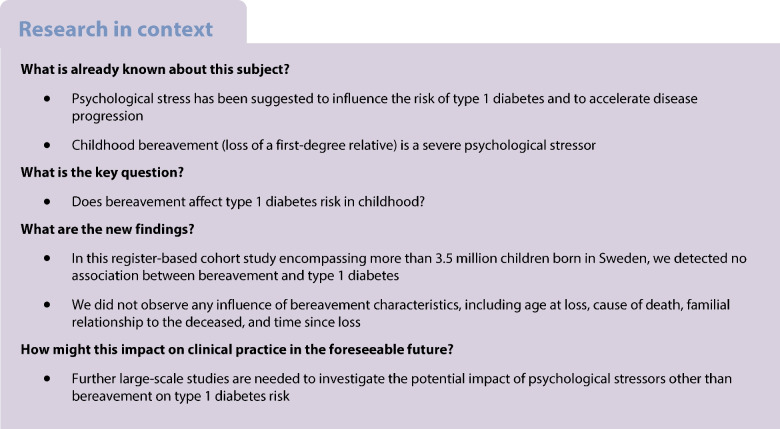


## Introduction

Type 1 diabetes is a chronic disease that is characterised by the autoimmune destruction of insulin-producing pancreatic beta cells. While genetic predisposition is an established risk factor for type 1 diabetes, the knowledge base on candidate environmental triggers and factors that may influence disease progression in childhood is rapidly evolving [1]. Psychological stress has previously been suggested to induce beta cell stress by increasing insulin demand [2, 3], and to increase the risk of islet autoimmunity by modulation of the immune response [4]. Childhood bereavement, i.e. the death of a parent or a sibling in childhood, constitutes a severe psychological stressor, with potentially far-reaching consequences for emotional well-being and psychiatric health [5–7]. However, the potential impact of bereavement on type 1 diabetes risk in childhood, and whether such effects are transient or of a more long-lasting nature, has not yet been fully elucidated.

Type 1 diabetes onset occurs at any time during childhood, but the incidence peaks during puberty [8], when rapid physical growth and substantial hormonal dynamics increase insulin resistance and demand. Moreover, the age at bereavement, and the developmental stage of the child, may influence how the child perceives and understands death [7]. The timing of the loss may thus have varying effects on type 1 diabetes development throughout childhood. Furthermore, previous Swedish register-based studies on childhood bereavement have reported that the health consequences may differ across causes of death, relationship to the deceased, and time since the loss [6, 9, 10]. Therefore, such factors may also be pertinent when assessing the association between loss and type 1 diabetes.

Several observational studies have suggested a link between psychological stressors, including childhood bereavement, illness of a family member or socioeconomic disadvantages, and type 1 diabetes incidence. However, the earliest studies were retrospective, questionnaire-based, or based on limited samples [11–15]. Two population-based register studies [16, 17], both originating from Danish national registers and using partly overlapping data, have reported inconclusive findings on type 1 diabetes risk after exposure to childhood adversities. While one study reported an age-dependent increased risk of type 1 diabetes [16], the other found no, or negligible, associations with type 1 diabetes risk [17]. A third Danish study [18], which specifically investigated the impact of childhood bereavement after the age of 5 years on type 1 diabetes in childhood or early adulthood, found an increased risk if the loss occurred after the age of 11 years. No assessment of variation by time since loss was performed.

Here, we aimed to investigate the effect of bereavement on the risk of childhood-onset type 1 diabetes, and to assess the potential influences of age at loss, cause of death, familial relationship to the deceased, and time since loss. To this end, we used prospectively and objectively collected data from national population and health registers in Sweden relating to more than 3.5 million children and their parents and siblings.

## Methods

### Study population

We obtained information on all children born in Sweden from 1987 to 2020 from the Total Population Register (*n*=3,704,422). The children were linked to their biological parents and siblings (full and half) using the Multi-Generation Register. Additional information on the children and their relatives was extracted from the Total Population Register, the Longitudinal Integrated Database for Health Insurance and Labour Market Studies, the National Patient Register (NPR), the Cause of Death Register and the Swedish Prescribed Drug Register. Linkages across registers were enabled through personal identification numbers, a unique 10-digit number that is assigned to all residents in Sweden at birth or immigration [19].

**Fig. 1:**
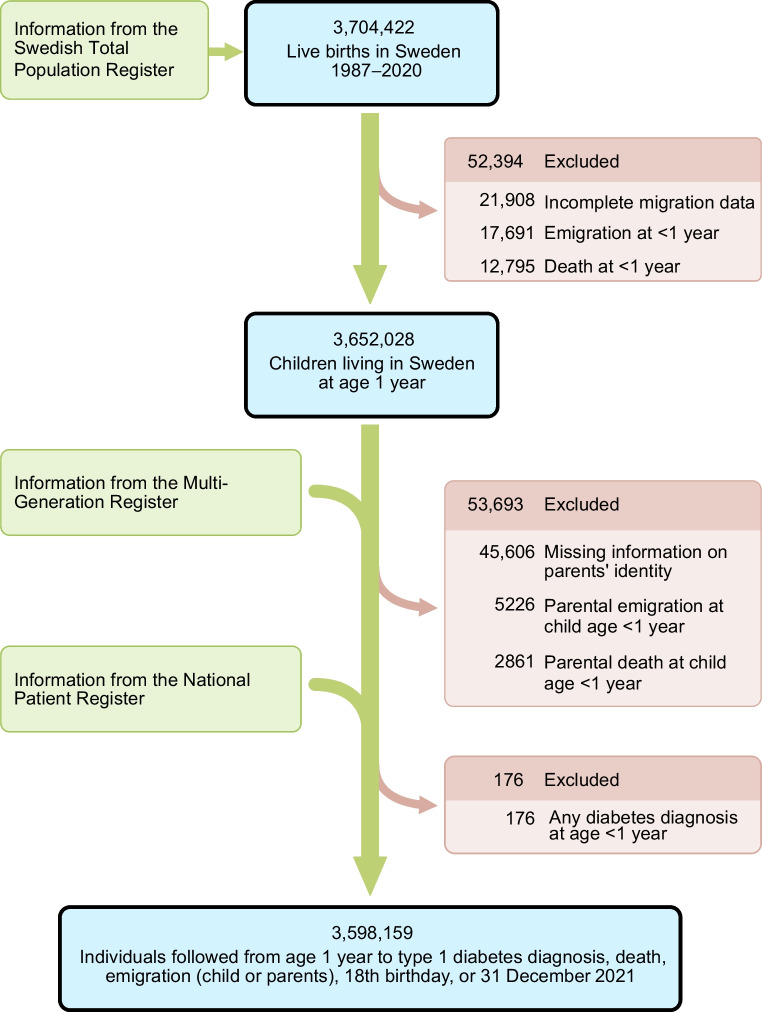
Flow chart of the study population

To enable the establishment of attachment relationships [20] and to avoid including events of neonatal diabetes, follow-up started at the age of 1 year. We excluded children who had died or emigrated before that age or who had incomplete migration data (Fig. 1). We also excluded children with missing information on the identity of their parents, and children with one or two parents who had died or emigrated before the start of follow-up. We further excluded children with any diabetes diagnosis (including neonatal diabetes or any other type of diabetes) recorded in the NPR before the start of follow-up. Any diabetes diagnosis before the start of follow-up was defined as having had an inpatient visit with the main diagnosis coded as 250 according to ICD-9 (http://www.icd9data.com/2007/Volume1/default.htm) or E10 or E11 according to ICD-10 (https://icd.who.int/browse10/2019/en). Our final study population comprised 3,598,159 children.

### Exposure

The main exposure, childhood bereavement, was defined as the death of a biological mother, father or sibling. The date of death of the parent or sibling was ascertained through the Cause of Death Register. We categorised age at loss as preschool (1–6 years), school age (7–12 years) or teenage (13–17 years). We categorised the main cause of death as illness (e.g. cardiovascular disease or cancer) or external causes of morbidity and mortality (including suicide, accidents, environmental exposures and homicides). External causes of morbidity and mortality were defined by ICD-9 codes E800–E999 and ICD-10 codes V01–Y98, while illness was defined as death by all other ICD-9 or ICD-10 codes. The familial relationship with the deceased was also investigated, and categorised as mother, father or sibling.

### Outcome

The main outcome was type 1 diabetes in childhood (1–17 years), defined as an inpatient main diagnosis (ICD-9 code 250 or ICD-10 code E10) in the NPR. The date of diagnosis was defined as the date of discharge.

Although ICD-9 does not have separate codes for the various types of diabetes, the risk of misclassification is low, because >98% of Swedish children aged 0–18 years who are diagnosed with diabetes have type 1 diabetes [21]. However, a previous study showed that prescription of insulin in the Swedish Prescribed Drug Register, which holds information on all dispensed medications in Sweden from July 2005 onwards, could be used to reliably assess the occurrence of type 1 diabetes in individuals aged 0–34 years [8]. To explore the validity of our outcome, we assessed the proportion of children defined as having type 1 diabetes who had at least one dispensed prescription of insulin (anatomical therapeutic chemical code A10A) at <18 years, and the proportion of children with one or more dispensed prescription of insulin at <18 years who did not have a type 1 diabetes diagnosis.

### Covariates

We obtained information on baseline parental covariates, including age, country of birth (categorised as Sweden or other), region of residence (categorised as Götaland, Svealand, Southern Norrland and Northern Norrland) and the population density of home municipality (calculated as the number of inhabitants per km^2^) from the Total Population Register. Baseline was defined as the year of birth of the child. Information on parental highest achieved education level (categorised as compulsory, secondary or university), disposable income (presented in quintiles) and marital status (categorised as married, not married but cohabiting with children, or single) was obtained from the Longitudinal Integrated Database for Health Insurance and Labour Market Studies, which collects sociodemographic information on all Swedish residents from 1990 onwards. Parental type 1 diabetes status was based on a diagnosis of type 1 diabetes (ICD-9 code 250 and/or ICD-10 code E10; main inpatient or outpatient diagnosis) in the NPR. Race as a concept is not used in Sweden, and information on race or ethnicity is not available in Swedish national registers and is therefore not included in our analyses [22]. All children in our cohort were born in Sweden.

Prior to the analysis phase, we created a directed acyclic graph using the DAGitty tool (available at http://www.dagitty.net) [23]. The directed acyclic graph (electronic supplementary material [ESM] Fig. 1) is a graphical presentation of the theoretical framework of the study, describes our prior assumptions on how bereavement may have a causal effect on child type 1 diabetes development, and further helps to identify potential confounders.

### Statistical methods

We used Cox proportional hazards models, with attained age as the timescale, to assess the association between the death of a family member and the risk of type 1 diabetes. Death of a family member was included in the models as a time-varying variable. An individual was classified as unexposed until the date of death of a family member, and was thereafter classified as exposed until the end of follow-up. If a child lost more than one family member during the study period, the first death was used to classify the exposure status. We did not evaluate the effect of multiple losses. The study individuals were censored at emigration (their own or parental), death, when the individual turned 18 years old, or at the end of follow-up (31 December 2021), whichever came first. To explore how the potential effect of losing a family member may vary by time since exposure to loss, we used restricted cubic splines (four knots at the 5th, 35th, 65th and 95th percentiles, respectively). A robust sandwich estimator of variance was used to account for the within-family correlations [24].

The models were adjusted for the potential confounders identified in our directed acyclic graph, i.e. the baseline variables year of birth of child, maternal age at delivery, parental country of birth, parental type 1 diabetes, region of residence and population density of the home municipality. However, information about parental type 1 diabetes was updated during follow-up and treated as a time-varying covariate. Less than 2% of our study population had missing information for the confounders, and complete-case analysis was performed. To examine the proportional hazards assumption, we used Schoenfeld residuals. These indicated that the assumption was violated for parental type 1 diabetes, and therefore a stratified Cox model allowing for different baseline hazards was used.

We further performed subgroup analyses by the sex of the child.

Analyses were performed using SAS version 9.4 (SAS Institute, USA) and R version 4.3 (The R Foundation, Austria) [25]. The study was approved by the Swedish Ethical Review Authority (DNR 2018/1697-31/1, with amendment 2021-03277).

## Results

### Validity of the outcome

According to our outcome definition based on the NPR, 18,817 children were diagnosed with type 1 diabetes (median age 9.1 years), of whom 98.8% (18,600) had a dispensed prescription of insulin. Of the 217 children diagnosed with type 1 diabetes who did not have an insulin prescription, 131 had already been censored by July 2005 when the Swedish Prescribed Drug Register was initiated. Overall, 19,610 children in our cohort had a dispensed prescription of insulin, of whom 5.2% (1010) did not have type 1 diabetes according to our outcome definition.

### Exposure to bereavement

In our final study population, 86,226 (2.4%) of the children were exposed to childhood bereavement, i.e. death of a family member. The median age at loss was 10.2 years. Of all deaths, 32.2% (27,801) were due to external causes. The most common loss was death of the father (44,620; 51.7%), followed by death of a sibling (21,860; 25.4%) and death of the mother (19,746; 22.9%). Baseline characteristics are presented in Table 1 and ESM Table 1. At birth, exposed children lived in less densely populated areas and had older parents than unexposed children. The parents of the exposed children were more often diagnosed with type 1 diabetes, had lower disposable income, lower education level, and were more often single than the parents of the unexposed children.

**Table 1:** Baseline characteristics of the study population, assessed at the year of birth, presented by exposure status

	Bereaved	Not bereaved
*N*	86,226	3,511,933
Birth year		
1987–1990	18,404 (21.3)	430,220 (12.3)
1991–1995	21,197 (24.6)	547,252 (15.6)
1996–2000	15,302 (17.7)	430,997 (12.3)
2001–2005	14,390 (16.7)	466,845 (13.3)
2006–2010	10,226 (11.9)	529,825 (15.1)
2011–2015	5265 (6.1)	549,965 (15.7)
2016–2020	1442 (1.7)	556,829 (15.9)
Girls	41,994 (48.7)	1,706,570 (48.6)
Mother born in Sweden	68,971 (80.0)	2,797,133 (79.6)
Missing data	245 (0.3)	20,443 (0.6)
Father born in Sweden	68,861 (79.9)	2,768,450 (78.8)
Missing data	349 (0.4)	54,146 (1.5)
Region of residence		
Götaland	40,180 (46.6)	1,662,589 (47.3)
Svealand	34,608 (40.1)	1,437,207 (40.9)
Southern Norrland	6312 (7.3)	225,012 (6.4)
Northern Norrland	5028 (5.8)	182,638 (5.2)
Missing data	98 (0.1)	4487 (0.1)
Population density (inhabitants per km^2^)	76 (29–380)	87 (34–717)
Missing data	98 (0.1)	4487 (0.1)
Maternal age at delivery (years)	30 (26–34)	29 (26–33)
Paternal age at delivery (years)	34 (29–39)	32 (28–36)
Maternal type 1 diabetes	616 (0.7)	16,462 (0.5)
Paternal type 1 diabetes	1063 (1.2)	20,580 (0.6)

Data are presented as *n* (%) or median (IQR)

Percentages are calculated based on numbers before excluding missing data

### No association between death of a family member and type 1 diabetes

During follow-up (median duration 15.3 years), the type 1 diabetes incidence rate was 51/100,000 person-years in the exposed children and 43/100,000 person-years in the non-exposed children. In total, 290 children were diagnosed with type 1 diabetes after experiencing the death of a family member, two were excluded from the adjusted analysis, and thus 288 children were included in our complete-case analysis dataset.

**Fig. 2:**
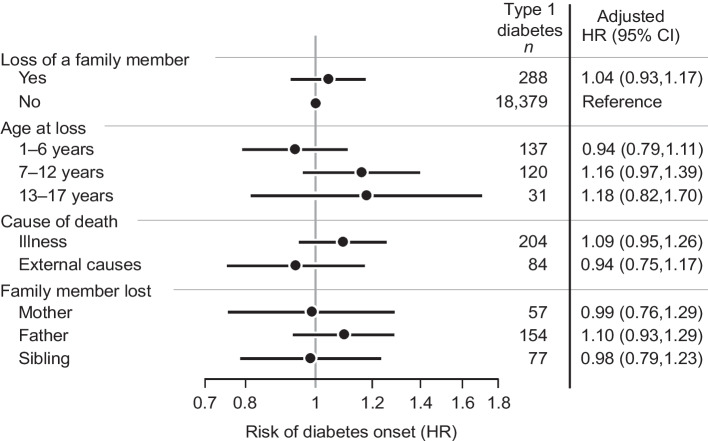
Adjusted HRs and 95% CI for type 1 diabetes in children exposed to bereavement, compared with unexposed children. The HRs were adjusted for year of birth of the child, maternal age at delivery, parental country of birth, parental type 1 diabetes, region of residence, and population density of the home municipality

**Fig. 3:**
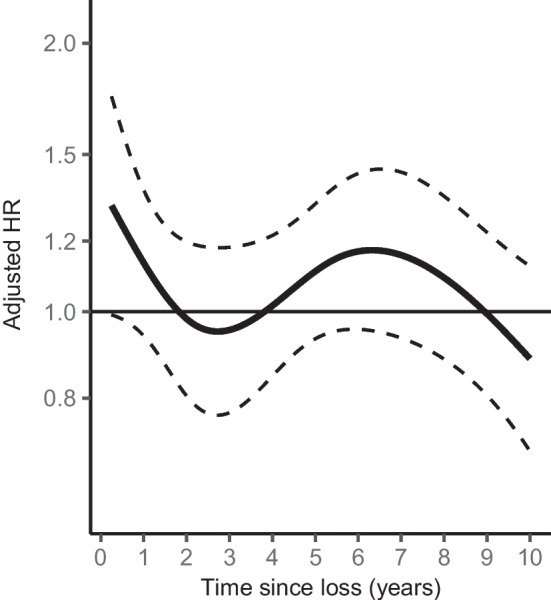
Adjusted time-varying HR and 95% CI for type 1 diabetes in children exposed to bereavement, compared with unexposed children. The HR was adjusted for year of birth of the child, maternal age at delivery, parental country of birth, parental type 1 diabetes, region of residence, and population density of the home municipality

We could detect no overall association between bereavement and type 1 diabetes (crude HR 1.10; 95% CI 0.98, 1.24; adjusted HR 1.04; 95% CI 0.93, 1.17) (Fig. 2). There was no influence of age at loss, cause of death, familial relationship to the deceased (Fig. 2) or time since loss (Fig. 3, *p*=0.096).

Finally, we could not detect any association between bereavement and type 1 diabetes when we performed a subgroup analysis by sex: adjusted HR for girls 1.15; 95% CI 0.96, 1.36; adjusted HR for boys 0.96; 95% CI 0.82, 1.13.

## Discussion

In the current study encompassing more than 3.5 million children, we detected no association between childhood bereavement and type 1 diabetes in childhood, or any influence of age at loss, cause of death, or familial relationship to the deceased. We also specifically investigated whether time since loss influenced the estimate, but found no evidence of such an association.

Strengths of our study include the population-based design, the large sample size with essentially complete follow-up, and the use of objectively and prospectively collected data, thereby enhancing the generalisability of our findings to similar populations and eliminating the risk of recall bias. Some limitations apply. First, although the death of a family member was objectively assessed and precisely dated, the child may have been exposed to increased psychological stress even before the loss if the death was due to illness or suicide. Second, we specifically investigated the emotional stress induced by the death of a biological family member, leaving unexplored other stressful life events such as the death of other close relatives, parental divorce or non-fatal disease in family members. Third, the sensitivity of type 1 diabetes diagnosis based on the NPR should be high as all children in Sweden with type 1 diabetes are initially hospitalised. However, the comparison with the prescription of insulin in our study indicated that some misclassification may be present. We think that this potential misclassification is non-differential and the risk is low and may have only a negligible effect on the estimated association. Fourth, even though our cohort comprised more than 3.5 million children, both the exposure and the outcome are relatively rare events. We may therefore have lacked adequate power in subgroup analyses by sex, or to investigate the impact of specific causes of death or age at loss. Lastly, our study population only encompassed children born in Sweden. Our results may not be generalisable to children born in countries with different incidence rates and trends in childhood-onset type 1 diabetes. Furthermore, under the Healthcare Act in Sweden, the children of parents with severe medical conditions should receive age-appropriate information and support from healthcare personnel to help them understand and process their parent’s condition and prognosis [26]. The financial impact of the loss of a parent may be partly alleviated by the child allowance, a monthly benefit distributed by the Swedish Pensions Agency to the surviving parent until the child turns 18 [27]. In case of severe disease and death of a sibling, parents can receive unlimited parental care allowance to care for their child in life and are eligible for sick pay after death [28]. In summary, our findings reflect the effect of childhood bereavement on type 1 diabetes incidence in a Nordic welfare state, and the external validity may be limited to other countries with similar healthcare and social support systems.

Our findings contrast with some previous reports on the relationship between exposure to psychological stress, defined as illness or death of a family member and/or socioeconomic disadvantages, and type 1 diabetes in childhood. These discrepancies may be partly due to methodological differences. Two population-based Danish studies, using partly overlapping register data, have previously investigated the impact of cumulative exposure to parental and psychosocial childhood adversities on type 1 diabetes risk in both childhood and early adulthood [16, 17]. While one of these reported a higher risk of type 1 diabetes in boys at age <11 years and in girls aged >16 years [16], the other reported an increased risk mainly in the small proportion of girls exposed to very high levels of adversity, with no increased risk of type 1 diabetes in boys [17]. Two smaller Swedish studies that indicated a link between exposure to an array of severe life stressors and type 1 diabetes in childhood were both questionnaire-based and performed in invited birth cohorts [14, 15].

Our overall findings align with the results of a Danish study that also focused on childhood bereavement and type 1 diabetes risk and included more than 1.7 million children born from 1980 to 2005 [18]. Similarly to our study, that study was based on national population and health registers, and observed no overall association between childhood bereavement and type 1 diabetes and no influence of cause of death or familial relationship to the deceased. The time since the loss was not explored. In contrast to our study, an increased risk of type 1 diabetes after loss of a family member at 11–17 years old was noted. However, their follow-up period extended into adulthood, while we aimed to investigate childhood-onset type 1 diabetes, and our results may not be fully comparable. Furthermore, the incidence rate of type 1 diabetes in childhood increased quite dramatically in Sweden during our study period [29], reaching an estimated age-standardised incidence of 41.4 cases per 100,000 person-years in individuals aged 0–19 years in 2021 [30]. In contrast, the Danish increase in type 1 diabetes incidence was less pronounced [31], reaching 25.4 cases per 100,000 person-years in 2021 [30]. Thus, our study included 18,817 children diagnosed with type 1 diabetes, compared with 6110 children and young adults in the Danish study, and the studies also reflect different baseline risks for type 1 diabetes in children.

In conclusion, our findings do not support a link between exposure to bereavement and type 1 diabetes in childhood.

## Supplementary Information

Below is the link to the electronic supplementary material.

ESM (PDF 91 KB)

## Data Availability

Restrictions apply to the availability of these data, which were used under licence and ethical approval and are not publicly available. However, data are available from the authors upon reasonable request and with written permission from the Swedish Ethical Review Authority, subject to legal contracts regarding the general data protection regulations (GDPR) and personal data processing agreements between Uppsala University and the recipient research entity.

## Abbreviation

NPR: National Patient Register

## References

[1] Rewers M, Ludvigsson J (2016) Environmental risk factors for type 1 diabetes. Lancet 387(10035):2340–2348. 10.1016/S0140-6736(16)30507-4 27302273 10.1016/S0140-6736(16)30507-4 PMC5571740

[2] Ludvigsson J (2006) Why diabetes incidence increases – a unifying theory. Ann NY Acad Sci 1079:374–382. 10.1196/annals.1375.058 17130582 10.1196/annals.1375.058

[3] Carlsson E, Frostell A, Ludvigsson J, Faresjö M (2014) Psychological stress in children may alter the immune response. J Immunol 192(5):2071–2081. 10.4049/jimmunol.1301713 24501202 10.4049/jimmunol.1301713

[4] Segerstrom SC, Miller GE (2004) Psychological stress and the human immune system: a meta-analytic study of 30 years of inquiry. Psychol Bull 130(4):601–630. 10.1037/0033-2909.130.4.601 15250815 10.1037/0033-2909.130.4.601 PMC1361287

[5] Bergman AS, Axberg U, Hanson E (2017) When a parent dies – a systematic review of the effects of support programs for parentally bereaved children and their caregivers. BMC Palliat Care 16(1):39. 10.1186/s12904-017-0223-y 28797262 10.1186/s12904-017-0223-y PMC5553589

[6] Rostila M, Berg L, Saarela J, Kawachi I, Hjern A (2019) Experience of sibling death in childhood and risk of psychiatric care in adulthood: a national cohort study from Sweden. Eur Child Adolesc Psychiatry 28(12):1581–1588. 10.1007/s00787-019-01324-6 30937545 10.1007/s00787-019-01324-6 PMC6861357

[7] Sood AB, Razdan A, Weller EB, Weller RA (2006) Children’s reactions to parental and sibling death. Curr Psychiatry Rep 8(2):115–120. 10.1007/s11920-006-0008-0 16539886 10.1007/s11920-006-0008-0

[8] Rawshani A, Landin-Olsson M, Svensson AM et al (2014) The incidence of diabetes among 0–34 year olds in Sweden: new data and better methods. Diabetologia 57(7):1375–1381. 10.1007/s00125-014-3225-9 24710965 10.1007/s00125-014-3225-9 PMC4052006

[9] Rostila M, Saarela JM (2011) Time does not heal all wounds: mortality following the death of a parent. J Marriage Family 73(1):236–249. 10.1111/j.1741-3737.2010.00801.x

[10] Rostila M, Berg L, Saarela J, Kawachi I, Hjern A (2017) Experience of sibling death in childhood and risk of death in adulthood: a national cohort study from Sweden. Am J Epidemiol 185(12):1247–1254. 10.1093/aje/kww126 28472250 10.1093/aje/kww126

[11] Sepa A, Ludvigsson J (2006) Psychological stress and the risk of diabetes-related autoimmunity: a review article. Neuroimmunomodulation 13(5–6):301–308. 10.1159/000104858 17709952 10.1159/000104858

[12] Sipetic S, Vlajinac H, Marinkovi J et al (2007) Stressful life events and psychological dysfunctions before the onset of type 1 diabetes mellitus. J Pediatr Endocrinol Metab 20(4):527–534. 10.1515/jpem.2007.20.4.527 17550217 10.1515/jpem.2007.20.4.527

[13] Karavanaki K, Tsoka E, Liacopoulou M et al (2008) Psychological stress as a factor potentially contributing to the pathogenesis of type 1 diabetes mellitus. J Endocrinol Invest 31(5):406–415. 10.1007/bf03346384 18560258 10.1007/BF03346384

[14] Nygren M, Carstensen J, Koch F, Ludvigsson J, Frostell A (2015) Experience of a serious life event increases the risk for childhood type 1 diabetes: the ABIS population-based prospective cohort study. Diabetologia 58(6):1188–1197. 10.1007/s00125-015-3555-2 25870022 10.1007/s00125-015-3555-2

[15] Lundgren M, Ellström K, Elding Larsson H, DiPiS study group (2018) Influence of early-life parental severe life events on the risk of type 1 diabetes in children: the DiPiS study. Acta Diabetol 55(8):797–804. 10.1007/s00592-018-1150-y 10.1007/s00592-018-1150-y PMC6060880 29752553

[16] Bengtsson J, Rieckmann A, Carstensen B, Svensson J, Jørgensen ME, Rod NH (2021) Trajectories of childhood adversity and type 1 diabetes: a nationwide study of one million children. Diabetes Care 44(3):740–747. 10.2337/dc20-1130 33495291 10.2337/dc20-1130

[17] Bengtsson J, Byberg S, Carstensen B et al (2020) Accumulation of childhood adversities and type 1 diabetes risk: a register-based cohort study of all children born in Denmark between 1980 and 2015. Int J Epidemiol 49(5):1604–1613. 10.1093/ije/dyaa138 33005951 10.1093/ije/dyaa138 PMC7746411

[18] Virk J, Ritz B, Li J, Obel C, Olsen J (2016) Childhood bereavement and type 1 diabetes: a Danish national register study. Paediatr Perinat Epidemiol 30(1):86–92. 10.1111/ppe.12247 26444317 10.1111/ppe.12247

[19] Ludvigsson JF, Almqvist C, Bonamy AK et al (2016) Registers of the Swedish total population and their use in medical research. Eur J Epidemiol 31(2):125–136. 10.1007/s10654-016-0117-y 26769609 10.1007/s10654-016-0117-y

[20] Howe N, Ross HS (1990) Socialization, perspective-taking, and the sibling relationship. Dev Psychol 26(1):160–165. 10.1037/0012-1649.26.1.160

[21] Ludvigsson JF, Ludvigsson J, Ekbom A, Montgomery SM (2006) Celiac disease and risk of subsequent type 1 diabetes: a general population cohort study of children and adolescents. Diabetes Care 29(11):2483–2488. 10.2337/dc06-0794 17065689 10.2337/dc06-0794

[22] European Commission (2021) Data collection in the field of ethnicity. Available from https://commission.europa.eu/system/files/2021-09/data_collection_in_the_field_of_ethnicity.pdf. Accessed 18 Nov 2024

[23] Textor J, van der Zander B, Gilthorpe MS, Liskiewicz M, Ellison GT (2016) Robust causal inference using directed acyclic graphs: the R package ‘dagitty.’ Int J Epidemiol 45(6):1887–1894. 10.1093/ije/dyw341 28089956 10.1093/ije/dyw341

[24] Lee EW, Wei LJ, Amato DA, Leurgans S (1992) Cox-type regression analysis for large numbers of small groups of correlated failure time observations. In: Klein JP, Goel PK (eds) Survival Analysis: State of the Art Nato Science Series E: Applied Sciences, Volume 211. Springer, Dordrecht, pp 237-247. 10.1007/978-94-015-7983-4_14

[25] R Core Team (2021) R: A language and environment for statistical computing. R Foundation for Statistical Computing, Vienna, Austria

[26] Bergman A-S, Hanson E (2014) Barn som är anhöriga när en förälder avlider: en kunskapsöversikt om effekt av metoder för stöd till barn. Available from https://www.diva-portal.org/smash/get/diva2:764517/FULLTEXT01.pdf. Accessed 22 Oct 2024. In Swedish

[27] Pensionsmyndigheten (2024) Survivor’s pension – financial support in the event of death. Available from https://www.pensionsmyndigheten.se/other-languages/english-engelska/english-engelska/survivors-pension-financial-support-in-the-event-of-death. Accessed 22 October 2024

[28] Försäkringskassan (2024) Vård av barn. Available from https://www.forsakringskassan.se/privatperson/foralder/vard-av-barn-vab. Accessed 22 October 2024. In Swedish

[29] Waernbaum I, Lind T, Möllsten A, Dahlquist G (2023) The incidence of childhood-onset type 1 diabetes, time trends and association with the population composition in Sweden: a 40 year follow-up. Diabetologia 66(2):346–353. 10.1007/s00125-022-05816-0 36264296 10.1007/s00125-022-05816-0 PMC9807495

[30] Ogle GD, James S, Dabelea D et al (2022) Global estimates of incidence of type 1 diabetes in children and adolescents: results from the International Diabetes Federation Atlas, 10th edition. Diabetes Res Clin Pract 183:109083. 10.1016/j.diabres.2021.109083 34883188 10.1016/j.diabres.2021.109083

[31] Carstensen B, Rønn PF, Jørgensen ME (2020) Prevalence, incidence and mortality of type 1 and type 2 diabetes in Denmark 1996–2016. BMJ Open Diabetes Research Care 8(1):e001071. 10.1136/bmjdrc-2019-001071 32475839 10.1136/bmjdrc-2019-001071 PMC7265004

