# Supplementary material for: Bereavement and type 1 diabetes in childhood: a register-based cohort study in Sweden
Source: Diabetologia. 2024 Dec 19;68(3):549–56. doi: 10.1007/s00125-024-06340-z (PMC11832628; doi:10.1007/s00125-024-06340-z)

Electronic supplementary material (ESM)

ESM Table 1. Baseline parental sociodemographic characteristics, presented by exposure status. No (%)

	Bereaved	Not bereaved
N	86,226	3,511,933
Maternal income quintiles [a]		
1 (Lowest)	14,476 (16.8)	600,871 (17.1)
2	14,640 (17.0)	602,035 (17.1)
3	14,232 (16.5)	602,730 (17.2)
4	11,517 (13.4)	605,494 (17.2)
5 (Highest)	11,541 (13.4)	605,083 (17.2)
Missing data	19,820 (23.0)	495,720 (14.1)
Paternal income quintile [a]		
1 (Lowest)	17,262 (20.0)	589,886 (16.8)
2	13,165 (15.3)	595,882 (17.0)
3	12,839 (14.9)	595,928 (17.0)
4	11,483 (13.3)	598,029 (17.0)
5 (Highest)	11,782 (13.7)	596,981 (17.0)
Missing data	19,695 (22.8)	535,227 (15.2)
Maternal highest education [b]		
Compulsory	16,026 (18.6)	376,296 (10.7)
Secondary	34,651 (40.2)	1,382,035 (39.4)
University	19,270 (22.3)	1,339,687 (38.1)
Missing data	16,279 (18.9)	413,915 (11.8)
Paternal highest education [b]		
Compulsory	17,732 (20.6)	434,461 (12.4)
Secondary	36,596 (42.4)	1,533,511 (43.7)

	Bereaved	Not bereaved
University	16,175 (18.8)	1,105,299 (31.5)
Missing data	15,723 (18.2)	438,662 (12.5)
Maternal marital status [b]		
Married	32,409 (37.6)	1,482,577 (42.2)
Cohabiting	28,185 (32.7)	1,410,268 (40.2)
Single	11,589 (13.4)	278,154 (7.9)
Missing data	14,043 (16.3)	340,934 (9.7)
Paternal marital status [b]		
Married	33,342 (38.7)	1,485,289 (42.3)
Not married but cohabiting with children	28,342 (32.9)	1,409,327 (40.1)
Single	10,178 (11.8)	226,553 (6.5)
Missing data	14,364 (16.7)	390,764 (11.1)

[a] Assessed the calendar year preceding the child's year of birth

[b] Assessed at the child's year of birth

Sociodemographic information from the Longitudinal Integrated Database for Health Insurance and Labour Market Studies is only available from 1990 onwards

ESM Figure 1. Directed Acyclic Graph of a theoretical framework to investigate the effect of loss of a family member due to death on type 1 diabetes. A directed edge (or "arrow") from one node to another represents a direct effect between these two nodes.

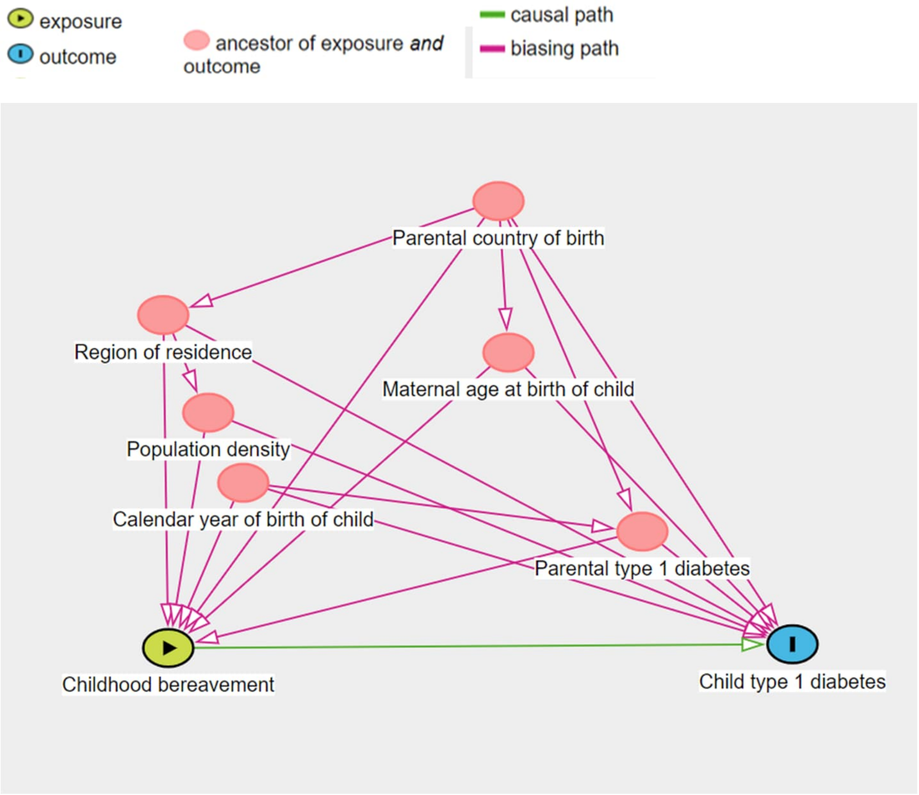

Supplement: Supplementary file 1 — ESM (PDF 91 KB) [file 125_2024_6340_MOESM1_ESM.pdf]
